# Di-(2-Ethylhexyl)-Phthalate (DEHP) Causes Impaired Adipocyte Function and Alters Serum Metabolites

**DOI:** 10.1371/journal.pone.0143190

**Published:** 2015-12-02

**Authors:** Nora Klöting, Nico Hesselbarth, Martin Gericke, Anne Kunath, Ronald Biemann, Rima Chakaroun, Joanna Kosacka, Peter Kovacs, Matthias Kern, Michael Stumvoll, Bernd Fischer, Ulrike Rolle-Kampczyk, Ralph Feltens, Wolfgang Otto, Dirk K. Wissenbach, Martin von Bergen, Matthias Blüher

**Affiliations:** 1 IFB *AdiposityDiseases*, University of Leipzig, Leipzig, Germany; 2 Department of Medicine, University of Leipzig, Leipzig, Germany; 3 Institute of Anatomy, University of Leipzig, Leipzig, Germany; 4 German Center for Diabetes Research (DZD), Leipzig, Germany; 5 Institute for Clinical Chemistry, Otto-von-Guericke-University Magdeburg, Magdeburg, Germany; 6 Department of Anatomy and Cell Biology, Martin Luther University Halle, Halle (Saale), Germany; 7 Department of Metabolomics, Helmholtz Centre for Environmental Research Leipzig, Leipzig, Germany; 8 Department of Proteomics, Helmholtz Centre for Environmental Research Leipzig, Leipzig, Germany; 9 Department of Biotechnology and Environmental Engineering, University of Aalborg, Aalborg, Denmark; East Tennessee State University, UNITED STATES

## Abstract

Di-(2-ethylhexyl)-phthalate (DEHP), an ubiquitous environmental contaminant, has been shown to cause adverse effects on glucose homeostasis and insulin sensitivity in epidemiological studies, but the underlying mechanisms are still unknown. We therefore tested the hypothesis that chronic DEHP exposure causes impaired insulin sensitivity, affects body weight, adipose tissue (AT) function and circulating metabolic parameters of obesity resistant 129S6 mice *in vivo*. An obesity-resistant mouse model was chosen to reduce a potential obesity bias of DEHP effects on metabolic parameters and AT function. The metabolic effects of 10-weeks exposure to DEHP were tested by insulin tolerance tests and quantitative assessment of 183 metabolites in mice. Furthermore, 3T3-L1 cells were cultured with DEHP for two days, differentiated into mature adipocytes in which the effects on insulin stimulated glucose and palmitate uptake, lipid content as well as on mRNA/protein expression of key adipocyte genes were investigated. We observed in female mice that DEHP treatment causes enhanced weight gain, fat mass, impaired insulin tolerance, changes in circulating adiponectin and adipose tissue Pparg, adiponectin and estrogen expression. Serum metabolomics indicated a general increase in phospholipid and carnitine concentrations. *In vitro*, DEHP treatment increases the proliferation rate and alters glucose uptake in adipocytes. Taken together, DEHP has significant effects on adipose tissue (AT) function and alters specific serum metabolites. Although, DEHP treatment led to significantly impaired insulin tolerance, it did not affect glucose tolerance, HOMA-IR, fasting glucose, insulin or triglyceride serum concentrations. This may suggest that DEHP treatment does not cause impaired glucose metabolism at the whole body level.

## Introduction

Obesity is the fastest growing health problem in Europe and worldwide. Overweight affects between 30% and 80% of adults in the WHO European Region and up to one third of children [1]. In addition to genetic factors, life style factors such as excessive caloric intake, high fat diets, and low physical activity contribute to obesity. However, there is also increasing evidence that environmental pollutants including endocrine-disrupting chemicals may contribute to the development of obesity and metabolic disorders [2]. Chronic exposure to endocrine-disrupting chemicals such as dioxins [3] bisphenol A [4] or phthalates [5,6] has been linked to an increased risk of developing insulin resistance or diabetes. Importantly, in the Prospective Investigation of the Vasculature in Uppsala Seniors Study of 1,016 elderly (age>70 years), Lind and coworkers demonstrated that several phthalate metabolites are related to diabetes prevalence, as well as to markers of insulin secretion and resistance [7].

Among those environmental factors, the plasticizer di(2-ethylhexyl)phthalate (DEHP), has been reported to induce glucose intolerance and alterations in hepatic glycogen content in rats. DEHP improves flexibility of polyvinyl chloride [8]. Humans are widely exposed to DEHP, because it is used in many daily products, including vinyl flooring, wall covering, plastic bags and covers, food containers, cosmetics, and toys [9]. Humans are exposed to these compounds through ingestion, inhalation and dermal exposure throughout their entire life, including intrauterine life. Although they are readily degraded, phthalates can cross the placenta and DEHP is consistently found in higher concentrations in children than in adults [10].

DEHP acts as an endocrine disruptor with toxic effects on reproductive and developmental processes [10]. Despite its widespread industrial use and presence as environmental contaminant, there are only a few reports on the effects of DEHP on adipose tissue function and insulin sensitivity [11–13,6].

In rats, DEHP promoted proliferation and induced hepatomegaly [14]. Liver enlargement was associated with a decrease in the activity of mitochondrial enzymes and modified the response to xenobiotics [14]. These effects on the liver are also reflected by alterations in the serum profiles of amino acids and phosphatidylcholines found in rats after DEHP-exposure [15]. In general, targeted and quantitative serum metabolomics have been proven to provide functional insights especially with regard to development and progress of metabolic diseases [16]. In another study, DEHP-fed rats had altered glucose tolerance associated with abnormal glucose intermediary metabolite content in liver and skeletal muscle; animals presented with a deficiency in glucose transport and a reduction in glycogen synthesis [17]. Moreover, Hong and co-workers hypothesized that oxidative stress caused by exposure to environmental chemicals plays a role in the pathogenesis of metabolic syndrome or type 2 diabetes mellitus by affecting insulin resistance [18].

In order to test the hypothesis that DEHP may impair insulin sensitivity, we exposed obesity resistant 129S6 mice to 2% DEHP for 10 weeks and assessed whole body insulin sensitivity and serum metabolomics. To investigate in more detail the role of DEHP on adipose tissue function *in vitro*, we used 3T3-L1 cells and measured proliferation, differentiation, glucose and lipid uptake as well as insulin sensitivity.

## Material and Methods

### Animals, diets, insulin tolerance test, and food intake

All animal experiments were performed in accordance with the *Guide for the Care and Use of Laboratory Animals* published by the US National Institutes of Health (NIH Publication No. 85–23, revised 1996) and were approved by the local authorities of the state of Saxony, Germany as recommended by the responsible local animal ethics review board (TVV08/09).

In 2007, breeding pairs from Taconic Farms, Inc. (129S6/SvEvTac; Hudson, New York, USA) were obtained and bred in our animal facility under standardized environmental conditions. Experiments were conducted on male and female of obesity resistant inbred 129S6 (F28) mice from our own Animal Laboratory at the University of Leipzig, Germany. Mice were kept acclimatized at 22°C ± 2°C and with a 12-hour light-dark cycle, as well as free access to food and water. At an age of 11 weeks, mice were randomly divided into two groups and fed ‘*ad libitum’* either a standard chow (control group, N = 16, 8 male/8 female) or the same standard diet supplemented with 0.05mg/kg body weight/day DEHP (DEHP group N = 16, 8 male/8 female) over a 10weeks experimental feeding period. This DEHP dose level is relevant to human exposure [19]. Food intake and body weight were carefully monitored and measured in both groups of animals throughout the entire experimental period. From twice a week measurements, food intake per animal, day and per body weight was calculated for the entire study period and shown for the measurements in weeks 5 and 6. The insulin tolerance test was performed after 6 weeks of treatment with standard chow alone or supplemented DEHP. Insulin (0.75U per kg body weight) was injected intraperitoneally. Tail vein blood was taken for glucose measurements at different time points at 0, 15, 30 and 60 minutes after insulin injection. i.p. GTT was performed in female mice after 8 weeks of treatment as previously described [20]. Homeostatic model assessment (HOMA) is a method for assessing β-cell function and insulin resistance (IR) from basal (fasting) glucose and insulin or C-peptide concentrations. HOMA-IR was calculated from basal (fasting) glucose and insulin concentrations from 6 female animals per experimental group at the end of observation period (10 weeks). At the end of observation period (10 weeks) whole body composition (fat mass, lean mass and total body water) was determined in awake mice by using nuclear magnetic resonance technology with EchoMRI700^™^ instrument (Echo Medical Systems, Houston, TX, USA) in control and DEHP treated mice. 6 animals per treatment group were measured. Data were analyzed by the manufacturer’s software.

Mice were placed in placed in metabolic cages (Tecniplast S.p.A. Buguggiate, Italy) to separate urine and feces over a period of 2 days. Mice had free access to food (standard chow) and water. At the end of 2 days urine samples were taken to quantify phthalates.

### Analytical procedures

Blood glucose values were determined from whole venous blood samples using an automated

glucose monitor (FreeStyle mini, Abbott GmbH, Ludwigshafen, Germany). Insulin, estradiol, progesteron and adiponectin serum concentrations were measured by ELISA using mouse standards according to the manufacturer’s guidelines (Mouse/Rat Insulin ELISA; CrystalChem. Inc, Downers Grove, IL), (Mouse/Rat Estradiol ELISA; Calbiotech Inc, Spring Valley, CA), (Mouse/Rat Progesterone ELISA; BioVendor, Karasek, Czech Republic) and (Mouse Adiponectin ELISA; AdipoGen Inc, Incheon, Korea). Serum concentrations of triglycerides and total cholesterol were analyzed by an automatic chemical analyzer in our Institute of Laboratory Medicine and Clinical Chemistry.

### Quantification of phthalates in urine

100μl urine aliquots were buffered with Na·ADA (sodium N-(2-acetamido)iminodiacetate, pH 6.6, final concentration 150mM), spiked with 13C4-labelled mono-(2-ethylhexyl)phthalate (MEHP), mono-(2-ethyl-5-hydroxyhexyl)phthalate (MEHHP), mono-(2-ethyl-5-oxohexyl)phthalate (MEOHP), mono-(2-ethyl-5-carboxypentyl)phthalate (MECPP), methylumbelliferone (MeUmb) and not isotopically labelled methylumbelliferyl glucuronide (final concentrations 9.5ng/ml each) and subjected to deglucuronation for 5h at 37°C (0.5U deglucuronidase / arylsulfatase, Helix pomatiae, Roche). Reactions were stopped by acidification (formic acid, final concentration 0.5M). Purification of samples was performed via solid phase extraction (Isolute C18 columns, 200 mg, Biotage). Eluted and vacuum-dried analytes were resuspended in 100μl 35% acetonitrile. For LC-MS/MS quantification 10 μl aliquots were separated on a UPLC System (UltiMate^™^ 3000 RSLC, Thermo scientific, MA, USA) via reversed phase chromatography (Acquity UPLC BEH C18, 1.7μm, 2.1mm x 100mm, Waters, Corporation, Milford, USA). Detection and quantification of analytes was achieved on a triple quadrupole mass spectrometer (Q-Trap 5500, AB Sciex) via electrospray ionisation (350°C, 4500 V, negative mode) using scheduled multi reaction monitoring (MRM) (phthalate analyte transitions. Absolute concentrations of analytes were calculated with respect to the known concentrations of the isotopically labelled standards and previously obtained calibration curves (quantification software Analyst, AB Sciex).

### Serum metabolome analysis

The metabolome analyses were carried out with the AbsoluteIDQ^®^ p180 Kit (Biocrates Life Science AG, Innsbruck, Austria). The kit identifies and quantifies 188 metabolites from 5 compound classes, namely acyl carnitines (40), proteinogenic and modified amino acids (19), glycerophospho- and sphingolipids (76 phosphatidylcholines, 14 lysophosphatidylcholines, 15 sphingomyelines), biogenic amines (19) and hexoses. 10μL serum samples were mixed with isotopically labeled internal standards were derivatized with phenylisothiocyanate and extracted. For LC-MS analysis of biogenic amines and amino acids and flow injection analysis-MS/MS measurements (FIA-MS/MS) were used two different dilutions. Both types of measurements were performed on a QTRAP mass spectrometer applying electrospray ionization (ESI) (ABI Sciex API5500Q-TRAP). The MS was coupled to an UPLC (Waters Acquity, Waters Corporation, Milford, USA). In case of LC-MS the metabolites were separated by an hyphenated reverse phase column (Agilent, Zorbax Eclipse XDB C18, 3.0 x 100mm, 3.5μm, Agilent Waldbronn, Germany) preceded with a precolumn (Security Guard, Phenomenex, C18, 4 x 3mm; Phenomenex, Aschaffenburg, Germany) applying a gradient. Identification and quantification were achieved by MRM standardized by applying spiked-in isotopically labelled standards in positive and negative mode, respectively. For calibration a calibrator mix consisting of 7 different concentrations was used. Quality controls were included for 3 different concentration levels. For FIA an isocratic method was used. The integrated MetIDQ software (Biocrates, Innsbruck, Austria) streamlines data analysis by automated calculation of metabolite concentrations providing quality measures and quantification.

Statistical analyses of metabolome data was based on the standard-evaluation of the Biocrates software and the manual adjustments, we performed pairwise group comparisons. For each group and each analyte covered by the Biocrates kit, we calculated the fold change together with the corresponding significance. The fold change is defined as the ratio between the mean of the analyte values of first group and the mean of the analyte values of the second group. The significance is calculated by a two-sample Wilcoxon test with the alternative hypothesis that the true difference in means is not equal to 0. Fold changes above 3/2 or below 2/3 with a corresponding p-value below 0.05 where considered significant.

### Cell culture

Mouse 3T3-L1 adipocytes cell line (genotype XX; ATCC^®^ CL-173^™^, American Type Culture Collection, Rockville, MD) were cultured with 0.01% DEHP for two days and differentiated into mature adipocytes as described previously [21]. Briefly, preadipocytes were grown to confluence in DMEM cell culture medium containing 25mM glucose (DMEM-H), 10% fetal bovine serum, and antibiotics (culture medium). After this period, cells were induced for 3 days in culture medium further supplemented with 1μM insulin, 0.5mM isobutylmethylxanthine, and 0.1μM dexamethasone. Subsequently, they were grown for 3 days in culture medium with 1μM insulin and for additional three to six more days in culture medium. Various effectors were added to cells starved in DMEM-H only for the indicated periods of time. At the time of the stimulation experiments, at least 95% of the cells had accumulated fat droplets.

### Measurements of glucose transport, palmitic acid incorporation and triglyceride content

In fully differentiated 3T3-L1 adipocytes, basal and insulin-stimulated glucose transport was measured using 2-deoxyglucose. Eight days after induction, 3T3-L1 adipocytes plated on 24 -well culture dishes were serum starved overnight. Cells were washed twice with Krebs-Ringer Hepes buffer at 37°C (20mmol/l HEPES, pH 7.4, 136mmol/l NaCl, 4.7mmol/l KCl, 1.25mmol/l MgSO4, 1.25mmol/l CaCl_2_) containing 0.1% BSA. The wash buffer was replaced with fresh Krebs-Ringer Hepes buffer and half of the wells were stimulated with 100nmol/l insulin (Roche, Basel, Schweiz) at 37°C for 30 minutes. The assay was initiated by adding 2-[^3^H]deoxy-D-glucose up to a final concentration of 0.5 μCi for 4 minutes at 37°C. The assay was terminated by adding 100μM 2-deoxyglucose (Sigma-Aldrich, St. Louis, MO, USA). After washing the cells with ice-cold PBS, adipocytes were detached from cell culture plates by using 0.1% SDS. Incorporated radioactivity was determined by scintillation counting and adjusted to protein concentration (BCA Protein Assay Kit, Thermo Fisher Scientific, Rockford, USA).

In addition, uptake of palmitic acid into 3T3-L1 cells was estimated by a [1-^14^C] palmitic acid based assay (Perkin Elmer, Massachusetts, USA). Fatty acid stock solution (8mM) was prepared by dissolving the sodium salt in BSA (3.3mM) solution. Eight days after induction of adipogenesis, 3T3-L1 adipocytes (on 24-well culture dishes) were washed once with Krebs-Ringer Hepes buffer at 37°C (20 mmol/l HEPES, pH 7.4, 120mmol/l NaCl, 6.0mmol/l KCl, 1.2mmol/l MgSO_4_, 1mmol/l CaCl_2,_ 1.2mmol/l KH_2_PO_4_) containing 0.1% BSA and incubated for 2 hours at 37°C in DMEM-H medium containing 1% BSA. After rewashing three times using Krebs-Ringer HEPES buffer containing 0.1% BSA, 1 ml of Krebs buffer (0.002% BSA) was applied to each well and radioactive uptake was started by adding ^14^C-palmitic acid up to a final concentration of 0.5μCi for 5 minutes at 37°C. Some wells were treated with 5μM of the fatty acid solution only to measure background activity. The assay was terminated by washing the cells with ice cold Krebs-buffer and detaching adipocytes from cell culture plates with 0, 1% SDS. Incorporated radioactivity was determined by scintillation counting and adjusted to protein concentration. Triglyceride content was determined using the LabAssay Triglyceride (WAKO Pure Chemicals, Kyoto, Japan).

### mRNA expression and western blot analysis

The mRNA expression of key genes, *adiponectin*, Phosphoinositide 3-kinase *(Pi3k*), insulin growth factor receptor 1 *(Igf1r)* and vesicle-associated membrane protein 4 (*Vamp4*) in adipose tissue function was measured using RT-PCR. RNA isolation and quantitative real-time PCR was performed as previously described [22]. In brief, mRNA expression was measured in a fluorescence temperature cycler using the TaqMan assay; fluorescence was detected on an ABI PRISM 7500 sequence detector (Applied Biosystems, Darmstadt, Germany). Total RNA was isolated using Trizol (Life Technologies, Grand Island, NY, USA), and 1 μg RNA was reverse transcribed with standard reagents (Life Technologies). From each RT-PCR, 1 μl was amplified in a 20 μl PCR reaction using Power Sybr Green Master Mix (ABI, Foster City, CA USA) according to the manufacturer’s protocol. Samples were incubated in the sequence detector for an initial denaturation at 95°C for 10 min, followed by 40 PCR cycles, each cycle consisting of 95°C for 15 s, 60°C for 30 s and 72°C for 32 seconds. mRNA level of *adiponectin*, phospho-inisitol 3 kinase *(Pi3k)*, insulin growth factor receptor 1 *(Igf1r)* and vesicle-associated membrane protein 4 *(V*a*mp4)* were determined. Specific mRNA expression was calculated relative to *36B4*, which was used as an internal control due to its resistance to glucose-dependent regulation [22]. Amplification of specific transcripts was confirmed by melting curve profiles at the end of each PCR. Expression levels are determined relative to endogenous control. All values are expressed as mean ± SEM if not indicated otherwise. Results from at least three experiments are shown. For *Western Blot analysis* 3T3-L1 adipocyte cultures were lysed by ultrasonication in 60 mM Tris-HCl, pH 6.8, containing 2% sodium dodecyl sulfate (SDS) and 10% sucrose. Cell lysates were diluted 1:1 in sample buffer (250 mM Tris-HCl, pH 6.8, containing 4% SDS, 10% glycerol, and 2% b-mercaptoethanol) and denatured at 95°C for 5 min. Protein concentration was assessed with the BCA protein assay (Pierbo Science, Bonn, Germany). Proteins (15 μg per lane) were separated by electrophoresis on a 12.5% SDS-polyacrylamide gel and transferred to nitrocellulose by electroblotting. Nonspecific binding sites were blocked with 5% dry milk for 45 min, then subsequently incubated with primary antibodies: mouse anti-adiponectin (Millipore Corporation, Billerica, MA; 1:2,000) and rabbit anti-phospho-PI3K (Phosphoinositide 3-kinase) (New England Biolabs GmbH, Frankfurt, Germany; 1:2000) at 4°C overnight. Proteins for detection of glucocorticoid receptor (anti-glucocorticoid-receptor, Glur, abcam, Cambridge, UK; 1:50,000) and estrogen receptor (anti-estrogen-receptor, Esr1, Abcam, Cambridge, UK; 1:500) were isolated from mouse adipose tissue and nonspecific binding sites were blocked with 5% bovine serum albumin (BSA) for 60 min. All proteins were detected by incubating with HRP conjugated secondary antibodies at a 1:3,000 dilution, (Dianova) at RT for 2 h and chemiluminescence kit (Amersham, Pharmacia, Freiburg, Germany). Integrated optical densities of the immunoreactive protein bands were measured with Gel Analyzer software (Media Cyberneties, Silver Spring, MD).

Subcutaneous (SC) and visceral adipose tissue was removed and homogenized with tissue-mill homogenizer (MM400Retsch GmbH, Haan, Germany) in RIPA buffer. Further procedures were performed as previously described [20] with antibodies raised against Peroxisome proliferator-activated receptor gamma (Ppary, 1:1.000, Cell Signaling Technology,#2430, Danvers, USA) and Adiponectin (Adiponectin, 1:1.000, Cell Signaling Technology,#2789, Danvers, USA). Equal protein loading was verified using mouse anti-D-glyceraldehyde-3-phosphate dehydrogenase antibody (GAPDH, Research Diagnostics, Flanders, The Netherlands; 1:3,000).

### Bromodeoxyuridine (BrdU) incorporation

BrdU incorporation was assessed in 3T3-L1 preadipocytes grown on glass coverslips. Cells were induced to differentiate for 24h, and then incubated with 10μM BrdU for 3h. Cells were fixed with ice-cold methanol for 10min. Fixed cells were incubated with 2M HCl for 1h at 37°C for DNA denaturation and then neutralized with 0.1M borate buffer (pH 8.5) for 15min. BrdU-labeled cells were visualized using an anti-BrdU antibody (Biomeda, Foster City, CA) and an Alexa Fluor^®^ 488 anti-mouse antibody (Invitrogen, Carlsbad, CA). BrdU-positive cells of 10 random microscopic fields were counted for each slide. BrdU staining for each sample were performed in triplicate.

### Statistics

Results are shown as mean ± SD unless stated otherwise. Differences between various treatments were analyzed by Student´s *t* tests. *P* values <0.05 were considered to be statistically significant.

## Results

### In vivo DEHP treatment causes increased body weight and alters insulin tolerance in female mice

**Fig 1:**
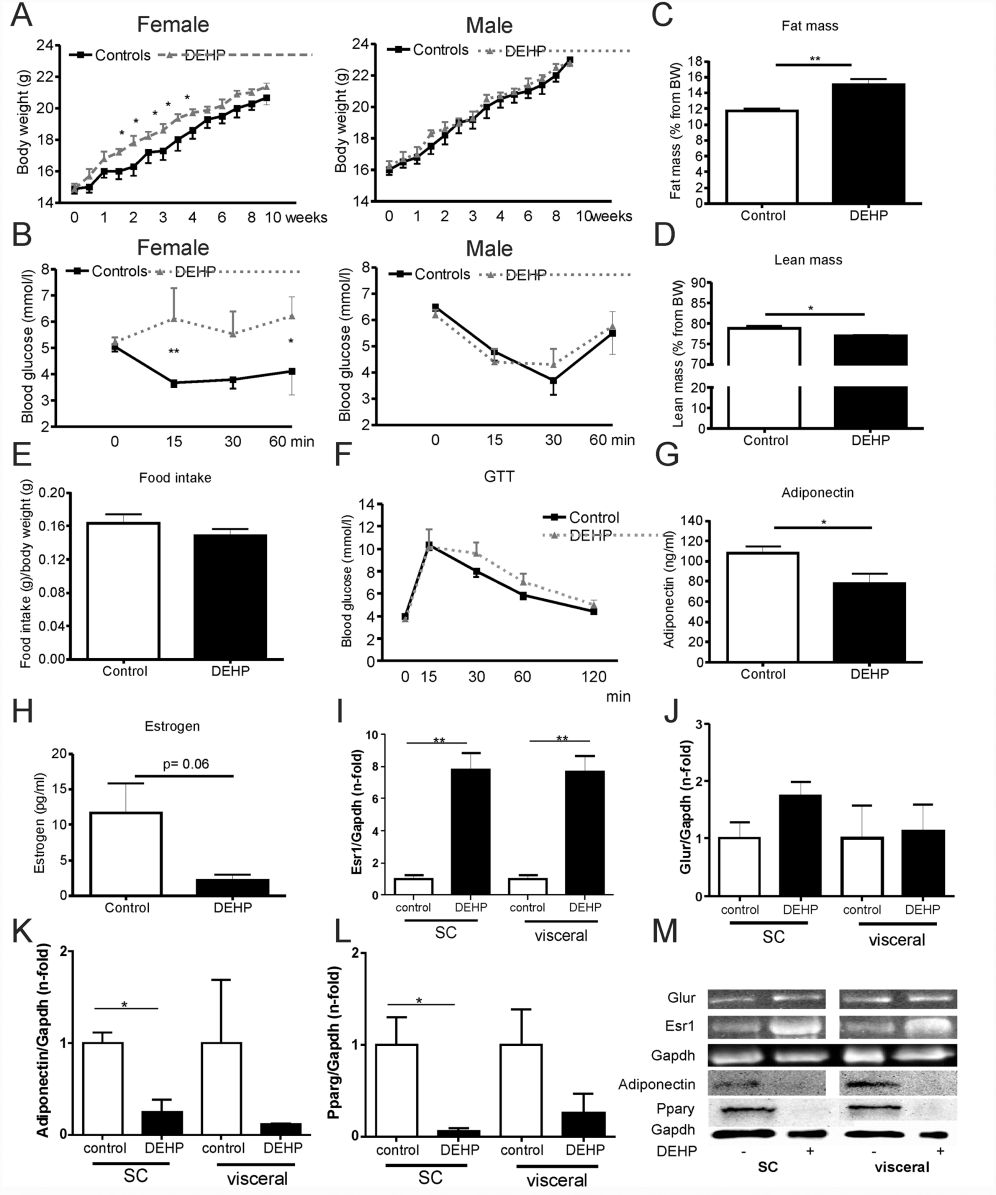
*In vivo* studies of 129S6 mice. **(A)** Body weight gain during treatment with DEHP **(B)** Insulin tolerance test of 129S6 mice (N = 8 controls, N = 8 DEHP treatment) after 6 weeks of DEHP intake **(C)** fat mass and **(D)** lean mass as percent of body weight was determined in awake female mice by using nuclear magnetic resonance technology with EchoMRI700 instrument (Echo Medical Systems, Houston, TX, USA) at the end of observation period (10 weeks). Data are presented as percentage of total body fat and lean mass from body weight. Results are expressed as means ± SE from at least 5 female animals per treatment group. **(E)** Food intake per animal, day and body weight calculated from food intake and body weight measurements in weeks 5 and 6 of treatment. **(F)** ipGTT was performed on 4-h-fasted after 8 weeks of DEHP treatment in female mice. Results are expressed as means ± SE from at least 5 female animals per treatment group. **(G)** Serum adiponectin and **(H)** estrogen concentrations were analyzed at the end of observation period (10 weeks) in female mice (n = 5 per treatment group). Data are presented as mean ± SE from at least 5 female animals per treatment group. **(I)** Western Blot quantification of estrogen receptor protein expression (Esr1), **(J)** glucocorticoid receptor (Glur), Adiponectin **(K)** and Ppary **(L)** in subcutaneous (SC) and visceral adipose tissue of female 129S6 mice (at least n = 3 per experimental group). **(M)** Representative images in adipose tissue of control and DEHP treated mice. Equal protein loading was verified using mouse anti-D-glyceraldehyde-3-phosphate dehydrogenase (Gapdh) antibody. The different degrees of significance (t-test with Welch correction) were indicated as follows in the graphs. *p<0.05; ** p< 0.01.

Female 129S6 mice treated with DEHP for 10 weeks showed a significantly greater weight gain and developed impaired insulin sensitivity (Fig 1A and 1B). In contrast, body weight dynamic and insulin sensitivity was not affected by DEHP in male 129S6 mice (Fig 1A and 1B). To further elucidate the body weight differences in females mice, we performed whole body composition analysis (fat mass, lean mass) in awake female mice by using nuclear magnetic resonance technology with EchoMRI700^™^ instrument. As shown in Fig 1, fat mass was significant higher and lean mass significant decreased in DEHP treated mice compared to control mice (Fig 1C and 1D). In addition, daily food intake was indistinguishable between female controls and female DEHP treated mice over the entire 10 weeks treatment period (data not shown), as well as in weeks 5 and 6 (Fig 1E). There was also no difference in daily food intake in male DEHP treated (0.17±0.06g/kg body weight/day) or control (0.18±0.05g/kg body weight/day) mice (p = 0.93). Impaired insulin tolerance in female DEHP treated mice was associated with a significant decrease in circulating adiponectin levels (Fig 1G), but no significant differences in fasted serum insulin or glucose levels (Table 1) compared with control mice. Despite indication of insulin resistance in the ipITT (Fig 1B) of female mice treated with DEHP, HOMA-IR and serum triglyceride concentrations did not reflect impaired insulin sensitivity in unchallenged fasted animals (Table 1).

**Table 1:** Phenotype of DEHP treated female 129S6 and control animals (n = 5 per group). Data obtained after 10 weeks of treatment are given as mean ± SEM.

Parameter	Control	DEHP	p-value
Fasting plasma glucose (mmol/l)	4.3 ± 0.1	4.3 ± 0.2	0.92
Fasting plasma insulin (ng/ml)	0.34 ± 0.04	0.46 ± 0.08	0.26
HOMA-IR	0.82 ± 0.15	1.07 ± 0.19	0.42
C-peptide (pmol/l)	705 ± 30	741 ± 24	0.36
Adiponectin (ng/ml)	107.7 ± 7.0	77.6 ± 9.9	**0.03**
Triglycerides (mmol/l)	1.5 ± 0.3	1.2 ± 0.1	0.11
Total Cholesterol (mmol/l)	2.5 ± 0.1	2.7 ± 0.1	0.22
Glycerol (nmol/ml)	681 ± 96	579 ± 34	0.27
Progesterone (ng/ml)	2.7 ± 0.3	6.7 ± 2.2	0.10
Estrogen (pg/ml)	11.7 ± 4.0	2.2 ± 0.8	0.06

Moreover, we found significantly reduced Pparg and adiponectin protein levels in SC adipose tissue of DEHP treated mice compared with control mice (Fig 1K, 1L and 1M).

To further elucidate potential mechanisms of increased body weight gain and fat mass in female 129S6 mice, we measured expression of estrogen receptor (Esr1) and glucocorticoid receptor (Glur) in subcutaneous (SC) and epigonadal (visceral) adipose tissue (Fig 1J). Importantly, higher DEHP exposure was associated with significantly 8-fold elevated Esr1 protein levels in both, SC and visceral adipose tissue compared to controls (p<0.01) (Fig 1I and 1M). A similar, but not significant trend towards higher expression upon DEHP exposure was observed for the glucocorticoid receptor (Fig 1J and 1M). Circulating serum estrogen (Fig 1H) and progesterone (Table 1) levels were decreased in DEHP treated mice. Serum triglycerides, cholesterol and serum glycerol levels (Table 1) were not affected by DEHP exposure in female mice.

### Primary transformation products of DEHP are at the same level as further oxidized DEHP metabolites

We confirmed a sufficient DEHP administration by detection of DEHP metabolites in urine (S1 Fig). In contrast to humans, where the secondary oxidation products MEHHP and MEOHP are tenfold higher abundant, the degradation pattern in mice is different. We observe similar levels of MEHP, the primary degradation product of DEHP in comparison to MEHHP and MEOHP. Interestingly, distribution of degradation products are identical for exposed and control animals, although at a different level (S1 Fig).

### The serum metabolome profile indicates significant changes in lipids and carnitines after DEHP exposure

**Fig 2:**
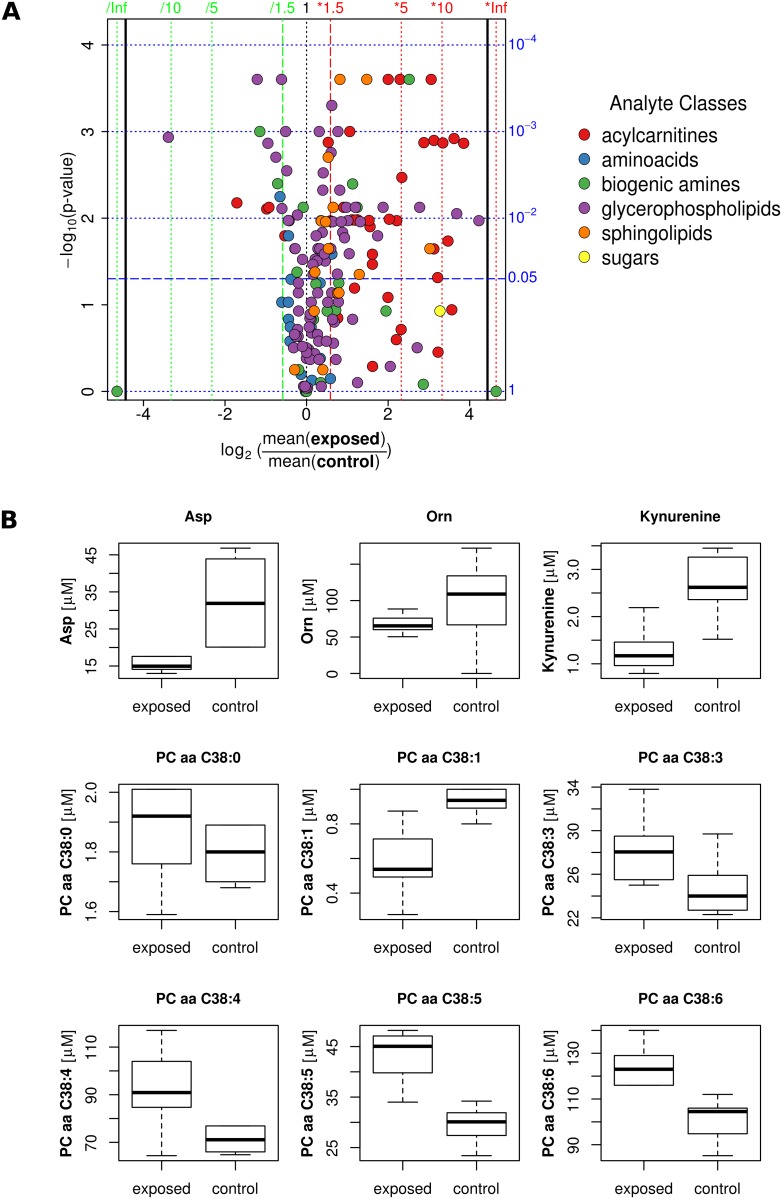
Serum metabolome profile indicates significant changes in lipids and carnitines. **(A)** Volcano plot (fold-change vs significance) of the comparison between metabolites of the exposed group and the control group of female 129S6 mice. The color code for different analyte classes is shown. **(B)** Box-and-whisker plot of selected metabolites. Medians, interquartile ranges (boxes) as well as minimal and maximal values (whiskers) are indicated. Significant differences between metabolites are marked with: *p<0.05, ** p<0.01, *** p<0.001, **** p<0.0001.

Overall distribution of metabolites is shown in the volcano plot (Fig 2A and S1 Table), indicating a general shift towards higher mean values for many lipids and carnitines. We found a clear trend both for lipids and carnitines, that the unsaturated forms are increased, whereas the saturated forms are not changed or even downregulated by DEHP exposure. As an example, the acyl-acyl forms of phosphatidylcholines containing 38 carbon atoms in their fatty acid chain are shown in more detail in Fig 2B. Similar behavior was found for carnitines and acyl-ethyl forms of phospholipids. DEHP treatment led to lowering of aspartate and kynurenine, but not ADMA and ornithine (Fig 2B).

### DEHP treatment enhances the proliferation of 3T3-L1 cells, but reduces cellular lipid content

**Fig 3:**
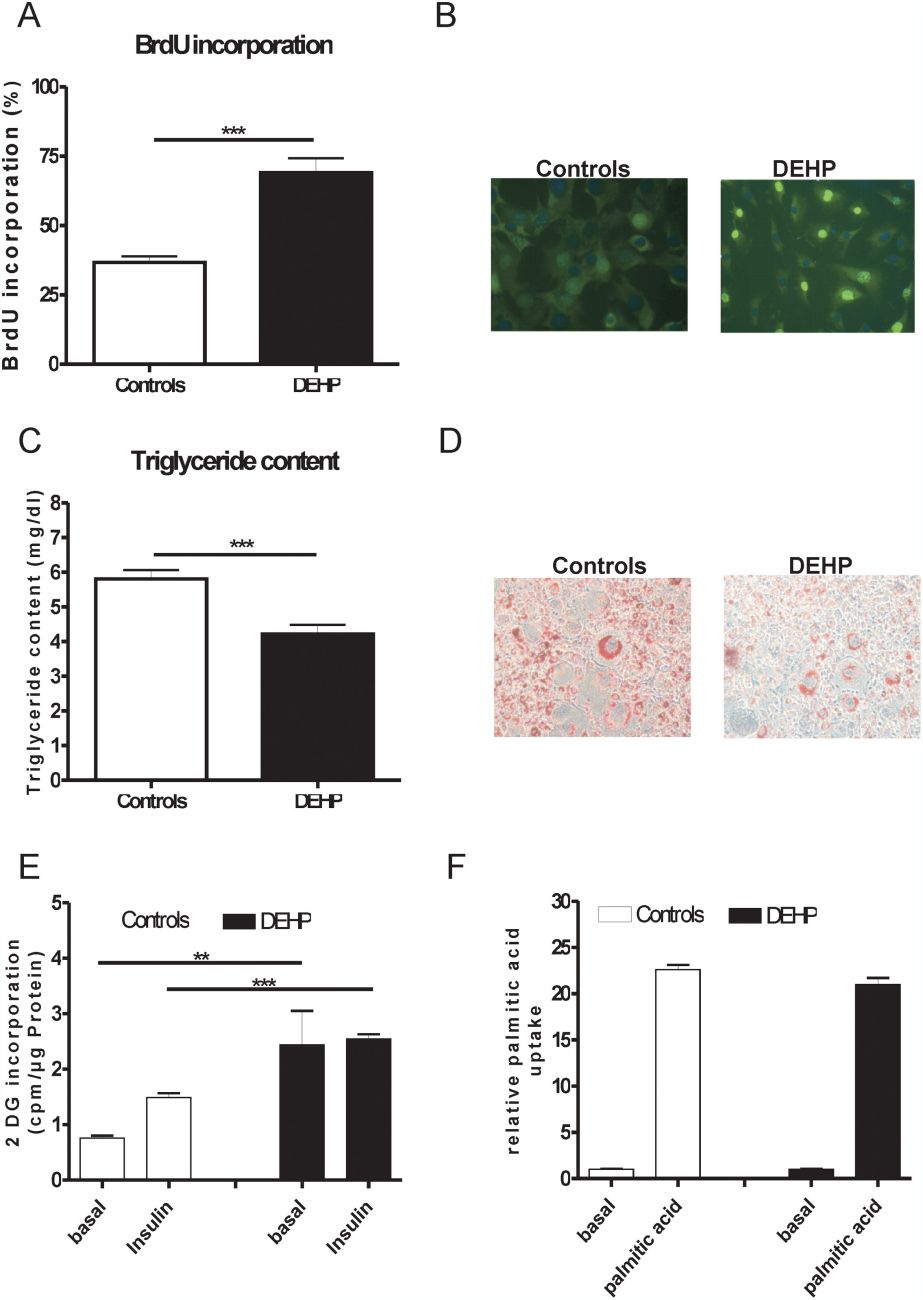
DEHP treatment causes enhanced proliferation rate, reduces lipid content, altered uptake of 2-deoxy-D [^14^C] glucose and similar palmitic acid uptake into 3T3-L1 adipocytes. **(A)** BrdU incorporation in 3T3-L1 adipocytes. BrdU incorporation was assessed in 3T3-L1 preadipocytes grown on glass coverslips. Cells were induced to differentiate for 24 h, and then incubated with 10 μM BrdU for 3 h. **(B)** BrdU staining- BrdU-labeled cells were visualized using an anti-BrdU antibody and an Alexa Fluor^®^ 488 anti-mouse antibody **(C)** Triglyceride content in mature 3T3-L1 cells **(D)** Oil Red O staining of 3T3-L1 adipocytes reveals reduced number of lipid droplet containing cells. 3T3-L1 cells were stained 8 days after induction (magnification 200x). **(E)** Basal and insulin stimulated uptake of 2-deoxy-D [^14^C] glucose was significantly increased in 3T3-L1 cells treated with DEHP. **(F)** Palmitic acid uptake in mature adipocytes with and without DEHP treatment. ** p<0.01, *** p<0.001.

To clarify in more detail the body composition changes under DEHP treatment, we performed *in vitro* analysis in 3T3-L1 cells. The BrdU incorporation study revealed a significant difference in the number of cells to incorporate BrdU between the control and DEHP treated 3T3-L1 cells in the mitotic clonal expansion phase (Fig 3A and 3B). BrdU incorporation was significantly elevated in DEHP treated cells indicating an enhanced proliferation rate (Fig 3). Mature 3T3-L1 cells treated with DEHP had lower cellular lipid content (Fig 3C and 3D).

### Basal glucose uptake is increased in DEHP treated 3T3-L1 cells

Treatment of 3T3-L1 cells with DEHP resulted in significantly increased basal glucose uptake and resistance to insulin stimulated glucose incorporation, suggesting that DEHP causes impaired insulin sensitivity at the cellular level (Fig 3E and 3F).

To investigate whether reduced cellular lipid content in response to DEHP treatment are explained by reduced fatty acid transport into adipocytes, we performed *in vitro* fatty acid uptake experiments. At day 8, control and DEHP treated 3T3-L1 adipocytes were starved overnight and incubated with ^14^C-labeled palmitate. However, we did not find differences in palmitate uptake between DEHP treated cells compared to controls (Fig 3F).

### Altered gene and protein expression profile in the DEHP treated adipocytes

**Fig 4:**
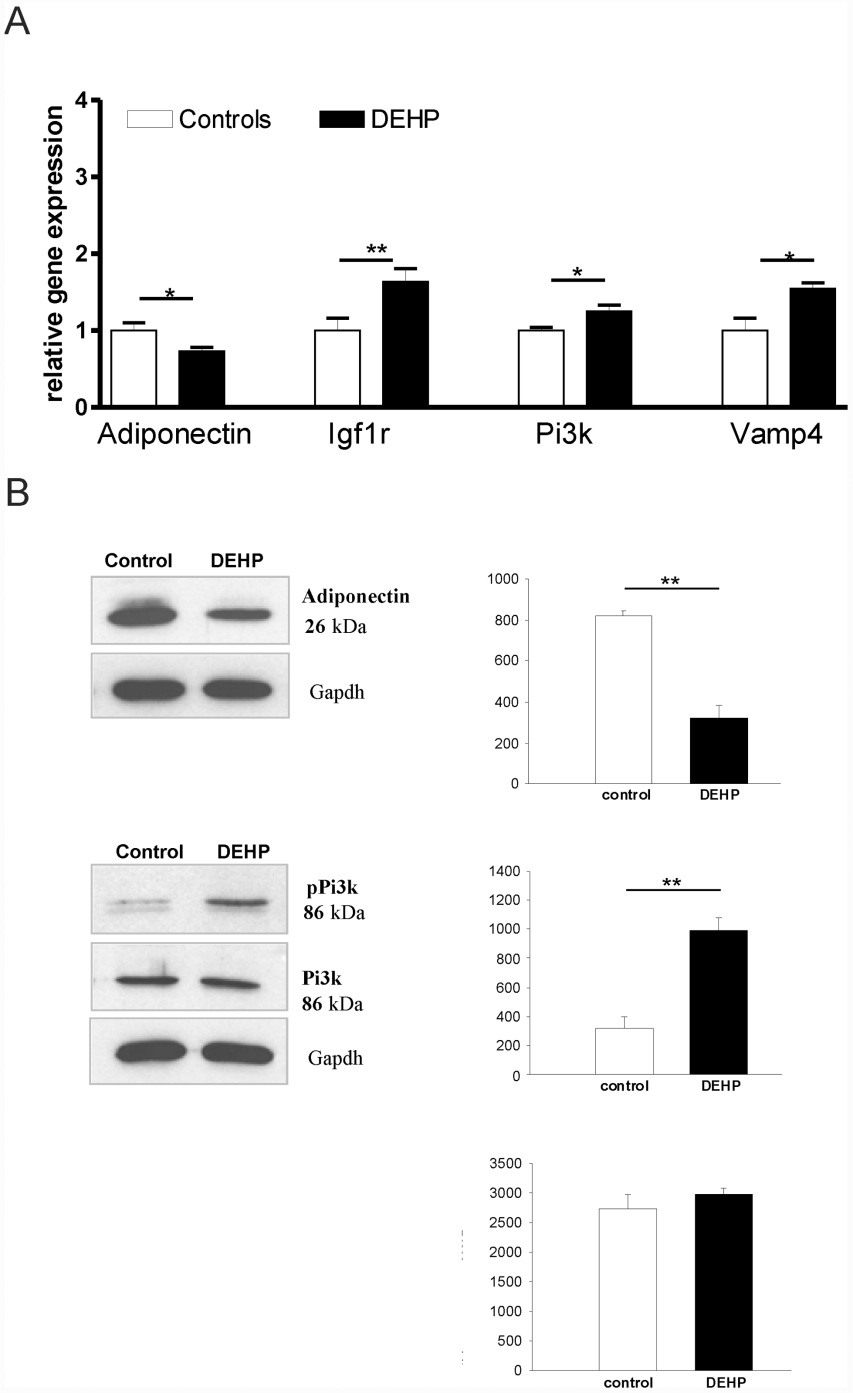
mRNA level and protein expression in mature 3T3-L1 adipocytes. mRNA expression of key genes **(A)** in adipose tissue function was performed using RT-PCR. **(B)** Western Blot analysis of adiponectin and phospho-phosphoinositol 3-kinase *(Pi3-k)* and quantification of protein level. * p<0.05, ** p<0.01

The expression analysis of key adipocyte genes revealed a significant suppression of *adiponectin* and elevation of phosphoinositol 3-kinase *(Pi3k)*, insulin like growth factor receptor 1 *(Igf1r)* as well as vesicle-associated membrane protein 4 (*Vamp4)*. Western blot analysis confirmed the adiponectin and phospho-Pi3k differences at the protein level (Fig 4A and 4B).

## Discussion

Phthalates are synthetic chemicals and ubiquitous environmental contaminants, to which humans are easily exposed, e.g. by leaching from food packaging materials [23]. DEHP is the most abundant phthalate in the environment [24]. Humans are exposed to these compounds through ingestion, inhalation and dermal exposure throughout their entire life. Although they are readily degraded, phthalates can cross the placenta and DEHP is consistently found in higher concentrations in children than in adults [1,3]. Strong evidence from experimental animal studies suggests that DEHP has many adverse effects on the nutritional and metabolic state [25]. In order to avoid a potential bias of body fat accumulation on impaired glucose homeostasis and insulin sensitivity in more obesity prone mouse models, we selected 129S6 mice—an obesity resistant mouse model—to study the effects of Di-(2-ethylhexyl)-phthalate on metabolism and adipose tissue function *in vivo*.

In the present work, we show that the effects of DEHP exposure on increasing body weight and body fat mass are female specific and independent of food intake. DEHP treatment and/or higher fat mass were associated with higher estrogen receptor (Esr1) expression in adipose tissue and a non-significant trend for lower circulating estrogen levels. Our results support data from a recent study in which DEHP induced estrogen receptor-α expression in a dose-dependent manner in human endometrial stromal cells *in vitro* [26]. However, it is unlikely that increased Esr1 expression in AT contributes to higher fat mass in DEHP treated mice, because mice lacking the ESR1 gene have more adipose tissue compared to wildtype mice [27] and human studies suggest an inverse relationship between AT Esr1 expression and BMI [28]. In humans, it has been further demonstrated that low adipose tissue ESR1 levels attenuate catecholamine resistance in SC fat cells of obese women thereby contributing to loss of SC and gain of visceral fat [29]. We confirm previous *in vitro* data that DEHP may cause reduced estradiol levels in mice [30].

Estrogens are recognized as key regulators of energy balance and glucose homeostasis since estrogen deficiency promotes visceral adiposity and insulin resistance in menopausal women [31]. Moreover, in experimental animals bilateral ovariectomy induces adipose tissue accumulation and glucose intolerance—a phenoptype, which can be prevented or reversed by 17β-estradiol treatment [32, 33]. The observed trend for reduced circulating estrogen levels in our study may therefore at least contribute to higher fat mass and impaired insulin tolerance in female mice and explain the sexual dimorphism in DEHP effects. Further studies are necessary to define the precise mechanisms how DEHP may cause decreased estrogen serum concentrations and increased AT estrogen receptor expression.

Interestingly, there was also a non-significant trend for higher Glur protein levels in AT after DEHP exposure. We cannot exclude that the effects of DEHP treatment on AT accumulation and changes in AT biology are mediated through enhanced glucocorticoid action. Glucocorticoid action on target tissues depends on circulating hormone levels, hormone-receptor interaction and intracellular prereceptor metabolism [34]. Increased sensitivity of AT to endogenous glucocorticoids may underly the observed AT phenotype in our study and requires further mechanistic studies. Noteworthy, we did not measure glucocorticoid serum concentrations upon DEHP treatment.

We further detected significantly lower circulating adiponectin and a significant down-regulation of Pparg and adiponectin in adipose tissue of DEHP treated mice. Lower adipose tissue *adiponectin* mRNA expression corresponds with lower protein level suggesting that DEHP may cause alterations in insulin sensitivity by reducing adiponectin expression. The phenotype of DEHP treated female 129S6 mice with higher body weight, fat mass and altered serum metabolites could be at least in part due to altered adipose tissue function, changes in adipokine secretion with reduced circulating adiponectin and down-regulation of Pparg.

Our data that DEHP reduces Pparg expression in subcutaneous adipose tissue are in contrast with a recent study [12]. Different mouse models (C3H/N in [12] versus 129S6 mice in our study) and differences in the DEHP exposure may explain these divergent data. Phthalate metabolites are known ligands to PPARs [7], receptors known to influence glucose homeostasis, impairments in PPAR-signaling pathways are most likely to contribute to the actions of phthalates on glucose metabolism and diabetes development. Both molecules, adiponectin and Pparg, are known to protect from insulin resistance and decreased adiponectin serum concentrations have been shown to be closely associated with insulin resistance in humans [35]. Because reduced adiponectin serum concentrations reflect adipose tissue dysfunction and may at least contribute to impaired whole body insulin sensitivity [36, 37], we provide now further evidence for a causality chain between DEHP treatment-induced adipose tissue dysfunction and impaired insulin sensitivity—at least suggested by insulin tolerance tests. However, fasting insulin, glucose, triglyceride serum concentrations as well as glucose tolerance and HOMA-IR were not significantly affected by DEHP treatment. Since all of these parameters showed a trend towards alterations consistent with impaired insulin sensitivity, we cannot exclude that the number of studied animals (n = 5) was too low to unravel significant DEHP effects on additional parameters of insulin resistance and impaired glucose metabolism. Due to the study size restriction provided by the local animal ethics review board, we could not increase the number of animals in the context of this study. Therefore, further studies, including a higher number of experimental animals and maybe using more sophisticated methods to assess insulin sensitivity such as euglycemic-hyperinsulinemic clamps should be performed.

Recently, studies in male albino Wistar rats suggested that DEHP induces insulin resistance in adipose tissue via increased reactive oxygen species production and lipid peroxidation that disrupts insulin signaling in adipose tissue and favors glucose intolerance [38]. We did not measure oxidative stress markers in adipose tissue of our mouse model and can therefore not exclude the proposed mechanism by Rajesh et al. [38]. Moreover, we did not detect an effect of DEHP treatment on body weight and increased fat mass in male 129S6 mice. In addition to the observed effects of DEHP on impaired insulin tolerance, we cannot exclude a DEHP effect on impaired beta cell function in female mice. Potential effects of chronic DEHP treatment on beta cells may be suggested by a trend of higher 30 and 60min glucose concentrations in response to the intraperitoneal glucose challenge (Fig 1F).

To further explore whether DEHP may have additional effects on circulating parameters of metabolism, we performed serum metabolomics studies. We found that DEHP treatment was associated with significantly higher serum concentrations of phosphatidylcholine diacyls (PCaa 38:3, PCaa 38:4, PCaa 38:5, PCaa 38:6) and significantly lower concentrations of phosphatidylcholine diacyl 38:1 and the amino acids aspartic acid and kynurenine. In general, we found a shift towards higher mean values for several phospholipids and carnitines, specifically the higher unsaturated molecule species: acyl-acyl and acyl-ethyl phospholipids and carnitines. These metabolites are known to be secreted into the circulation by adipose tissue and have been associated with the loss of body fat in a humans undergoing bariatric surgery [39]. Moreover, in genetically obese mice, phosphatidylcholine diacyls C38:4 and C38:5 have been recently found to be significantly lower compared to lean control mice [40]. These data seem to be in part contradictory to our metabolomics results, particularly since DEHP treated mice had significantly higher fat mass compared to controls at the time point of metabolite analyses. Based on the metabolite profile differences between our study and previous reports [39, 40], we hypothesize that DEHP may alter serum concentrations of distinct phospholipids and carnitines via adipose tissue independent mechanisms.

Several phospholipids and carnitines have been described to be regulated in an age and/or gender dependent manner in humans [41]. However, one limitation of our study is that we were not able to adjust for potential gender and age effects.

In contrast to previous rat DEHP exposure studies, we found a decrease of aspartate upon DEHP treatment, suggesting that the effects of DEHP maybe species specific [42]. Kynurenine serum concentrations are significantly lower upon DEHP treatment and its associated higher fat mass in our study. In contrast to these data, human studies found consistently higher kynurenine concentrations in individuals with obesity and type 2 diabetes [43, 44, 45, 46]. These divergent findings suggest that DEHP induced increase in fat mass may cause different alterations in the circulating metabolome than AT accumulation caused by other mechanism.

We further sought to define the effects of DEHP on adipose tissue in an *in vitro* model, the 3T3-L1 cells. In this model system, we demonstrate that the plasticizer DEHP has significant enhancing effects on adipocyte proliferation, but reduces cellular lipid content. These results confirm previous findings that DEHP elevates the proliferation rate in the hepatocyte cell line (HepG2) and extend it to an adipocyte model [46]. Recently, Chen et al. (2013) provided experimental evidence that activation of the PI3K-AKT-mTOR signaling pathway promotes DEHP-induced proliferation in Hep3B cells [47]. In accordance with these data, we find significant activation of phosphoinositide 3-kinase in 3T3L1 cells induced by DEHP *in vitro*. We therefore hypothesize that increased proliferation of 3T3L1 cells may be induced by activation of the PI3K signaling pathway. Our data further support previous findings that DEHP promotes adipogenic differentiation of murine mesenchymal stem cells (MSC, C3H/10T1/2) [48]. At the organism level, theoretically higher energy demand for increased adipose tissue proliferation upon DEHP treatment may be covered by reduced lean body mass.

We found that DEHP exposure led to significantly decreased cellular lipid content in 3T3-L1 cells. In principle, reduced lipid droplet size in adipocytes could be due to decreased triglyceride synthesis, decreased glucose or fatty acid uptake and/or increased lipolysis. Surprisingly, both, basal and insulin stimulated glucose uptake were significantly increased by DEHP in 3T3-L1 cells. This result is in accordance with increased glucose uptake into rat adipocytes after DEHP stimulation [38]. We further determined palmitic acid incorporation into 3T3-L1 cells by radioactivity assay. Since palmitic acid uptake was not affected by DEHP treatment, lower lipid content in DEHP-treated 3T3-L1 cells are most likely due to increased lipolysis. From rat studies it is known that DEHP intake effects lipolysis and LPL activity, with regard to the potential contribution to the known fat-lowering effect of this plasticizer [49, 50]. However, we did not measure *in vitro* lipolysis directly. Importantly, DEHP treatment of 3T3L1 cells in our study caused cellular changes, which closely resembles the phenotype of 3T3-L1 adipocytes that overexpress a dominant negative PPARγ mutant [51]. Because, DEHP treatment significantly downregulated PPARγ in 3T3L1 cells, our data suggest that PPARγ dependent mechanisms underly the observed lower triglyceride content and altered glucose uptake—similar to that shown for an experimental deletion of PPARγ in 3T3L1 cells [51]. Reduced PPARγ in 3T3L1 cells further caused changes in the abundance of mRNAs for several key enzymes that contribute to triglyceride and free fatty acid metabolism as well as the amounts of *GLUT4*, *insulin receptor*, *insulin receptor substrate* (*IRS*), and *C/EBPα* mRNAs [51]. Taken together, data from our 3T3-L1 DEHP treatment studies strongly suggest that reduced PPARγ (and subsequent gene expression changes) caused the observed alterations in cellular triglyceride content and glucose transport.

On the other hand, several other mechanisms could be involved in reduced lipid droplet size of DEHP treated 3T3-L1 cells. We therefore tested the hypothesis that lipid content alterations are due to changes in vesicle formation. In comparison with control cells, DEHP treated cells had significant higher vesicle-associated membrane protein 4 (*Vamp4)* expression, indicating an impaired vesicle formation process [52]. Further studies are required to test the hypothesis that DEHP directly affects *Vamp4* expression. Differences of VAMP4 protein expression has been recently shown to influence the rate of lipid droplet fusion and the size of lipid droplets [52]. Transfection of NIH-3T3 cells with *Vamp4* siRNA decreased the size of the lipid droplets and lipid fusion rate [52]. Moreover, we found increased Igf1r expression following DEHP treatment of 3T3L1 cells to be associated with smaller lipid droplet size. We previously demonstrated that conditional IGF-1R inactivation results in increased adipocyte size and adipose tissue mass suggesting that intact Igf1r signaling in adipocytes maybe important in the regulation of lipid storage capacity [53]. In the light of these data, we propose that DEHP-associated increase in Igf1r expression contributes to smaller lipid droplet size in 3T3L1 cells.

Taken together our findings suggest that DEHP may cause adipocyte dysfunction (reflected by altered adiponectin secretion and decreased Pparg protein expression), which could contribute to increased fat mass, impaired insulin tolerance and changes to serum metabolites.

## Conclusions

In conclusion, our data provide evidence that chronic DEHP treatment causes increased body weight, fat mass and altered serum metabolites in a gender specific manner, which could be mediated by a DEHP-induced increase in estrogen receptor expression and reduced Pparg expression in adipose tissue. Although, DEHP treatment led to significantly impaired insulin tolerance, it did not affect glucose tolerance, HOMA-IR, fasting glucose, insulin or triglyceride serum concentrations. This may suggest that DEHP treatment does not cause impaired glucose metabolism at the whole body level.

## Supporting Information

S1 Fig Box-and-whisker plot of urinary DEHP metabolites. Box-and-whisker plot of the (log_10_ scaled) concentrations of the four urinary DEHP metabolites MEHP, MEHHP, MEOHP and MECPP in female DEHP-fed (n = 13; black) and control (n = 5; white) animals. Medians, interquartile ranges (boxes) as well as minimal and maximal values (whiskers) are indicated. Observable differences are highly significant; performing one-sided Mann-Whitney U tests (with the alternative hypothesis c_treated_ > c_untreated_) leads to the rejection of the null-hypothesis (p < 5 x 10^−4^) for all metabolites.
(TIFF)

S1 Table List of serum metabolome profile. List of serum metabolome profile indicates significant changes in lipids and carnitines after DEHP exposure.
(DOCX)
