# Supplementary figures and images for: Di-(2-Ethylhexyl)-Phthalate (DEHP) Causes Impaired Adipocyte Function and Alters Serum Metabolites
Source: PLoS One. 2015 Dec 2;10(12):e0143190. doi: 10.1371/journal.pone.0143190 (PMC4668085; doi:10.1371/journal.pone.0143190)

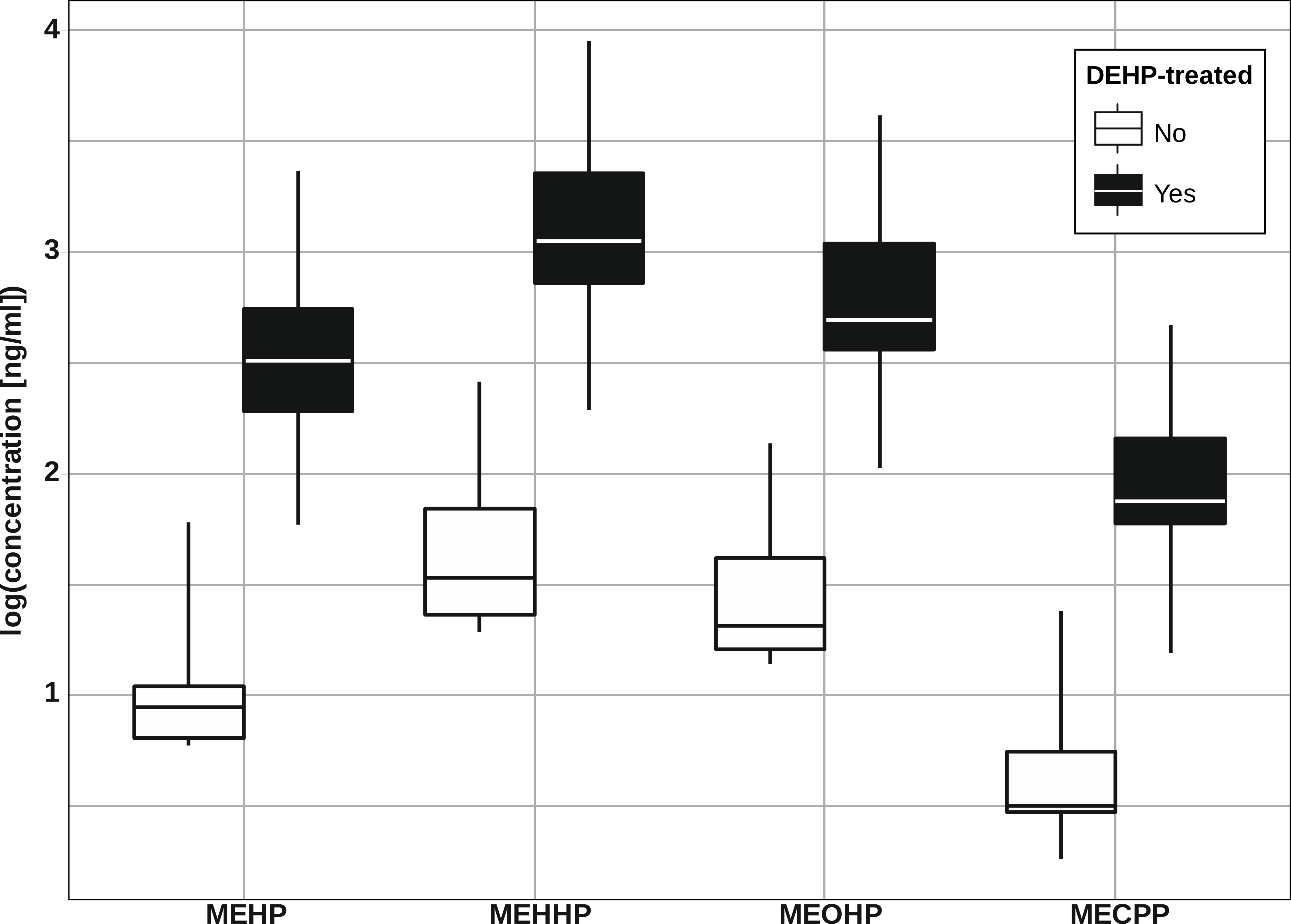

Supplement: S1 Fig — Box-and-whisker plot of the (log10 scaled) concentrations of the four urinary DEHP metabolites MEHP, MEHHP, MEOHP and MECPP in female DEHP-fed (n = 13; black) and control (n = 5; white) animals. Medians, interquartile ranges (boxes) as well as minimal and maximal values (whiskers) are indicated. Observable differences are highly significant; performing one-sided Mann-Whitney U tests (with the alternative hypothesis ctreated > cuntreated) leads to the rejection of the null-hypothesis (p < 5 x 10^−4^) for all metabolites. (TIFF) [file pone.0143190.s001.tiff]
