# Supplementary material for: Di-(2-Ethylhexyl)-Phthalate (DEHP) Causes Impaired Adipocyte Function and Alters Serum Metabolites
Source: PLoS One. 2015 Dec 2;10(12):e0143190. doi: 10.1371/journal.pone.0143190 (PMC4668085; doi:10.1371/journal.pone.0143190)
Supplement: S1 Table — List of serum metabolome profile indicates significant changes in lipids and carnitines after DEHP exposure. (DOCX) [file pone.0143190.s002.docx]

**Supplemental Table T1**. List of serum metabolome profile indicates significant changes in lipids and carnitines after DEHP exposure

| **analyte** | **class** | **Fold**  **change (mean)** | **p (U, paired)** | **p-adj (fdr)** | **def** |
| --- | --- | --- | --- | --- | --- |
| C0 | acylcarnitines | 1,50 | 0,01075 | 0,03097 | 1,00 |
| C10 | acylcarnitines | 3,99 | 0,08221 | 0,13776 | 1,00 |
| C10:1 | acylcarnitines | 3,06 | 0,03403 | 0,06832 | 1,00 |
| C10:2 | acylcarnitines | 1,45 | 0,00133 | 0,01154 | 1,00 |
| C12 | acylcarnitines | 2,94 | 0,01254 | 0,03482 | 1,00 |
| C12-DC | acylcarnitines | 1,70 | 0,14223 | 0,20728 | 1,00 |
| C12:1 | acylcarnitines | 2,26 | 0,06421 | 0,11374 | 1,00 |
| C14 | acylcarnitines | 8,71 | 0,02264 | 0,05074 | 1,00 |
| C14:1 | acylcarnitines | 4,90 | 0,00025 | 0,00581 | 1,00 |
| C14:1-OH | acylcarnitines | 14,45 | 0,00136 | 0,01154 | 1,00 |
| C14:2 | acylcarnitines | 8,31 | 0,00025 | 0,00581 | 1,00 |
| C14:2-OH | acylcarnitines | 8,72 | 0,00127 | 0,01154 | 1,00 |
| C16 | acylcarnitines | 3,06 | 0,02607 | 0,05573 | 1,00 |
| C16-OH | acylcarnitines | 4,99 | 0,19273 | 0,25977 | 1,00 |
| C16:1 | acylcarnitines | 2,08 | 0,001 | 0,01154 | 1,00 |
| C16:1-OH | acylcarnitines | 10,14 | 0,00135 | 0,01154 | 1,00 |
| C16:2 | acylcarnitines | 11,06 | 0,01837 | 0,04555 | 1,00 |
| C16:2-OH | acylcarnitines | 12,26 | 0,0012 | 0,01154 | 1,00 |
| C18 | acylcarnitines | 4,60 | 0,25265 | 0,32409 | 1,00 |
| C18:1 | acylcarnitines | 4,00 | 0,00025 | 0,00581 | 1,00 |
| C18:1-OH | acylcarnitines | 9,28 | 0,04852 | 0,09116 | 1,00 |
| C18:2 | acylcarnitines | 5,05 | 0,00338 | 0,02246 | 1,00 |
| C2 | acylcarnitines | 0,82 | 0,02248 | 0,05074 | 1,00 |
| C3 | acylcarnitines | 0,51 | 0,00783 | 0,03097 | 1,00 |
| C3-DC (C4-OH) | acylcarnitines | 1,88 | 0,00749 | 0,03097 | 1,00 |
| C3-OH | acylcarnitines | 11,80 | 0,11417 | 0,17826 | 1,00 |
| C3:1 | acylcarnitines | 2,89 | 0,01063 | 0,03097 | 1,00 |
| C4 | acylcarnitines | 0,31 | 0,00666 | 0,03097 | 1,00 |
| C4:1 | acylcarnitines | 4,63 | 0,01069 | 0,03097 | 1,00 |
| C5 | acylcarnitines | 0,53 | 0,00749 | 0,03097 | 1,00 |
| C5-DC (C6-OH) | acylcarnitines | 2,28 | 0,01052 | 0,03097 | 1,00 |
| C5-M-DC | acylcarnitines | 2,16 | 0,01046 | 0,03097 | 1,00 |
| C5-OH (C3-DC-M) | acylcarnitines | 1,28 | 0,01069 | 0,03097 | 1,00 |
| C5:1 | acylcarnitines | 2,38 | 0,01046 | 0,03097 | 1,00 |
| C5:1-DC | acylcarnitines | 2,28 | 0,01052 | 0,03097 | 1,00 |
| C6 (C4:1-DC) | acylcarnitines | 0,69 | 0,01598 | 0,04073 | 1,00 |
| C6:1 | acylcarnitines | 4,08 | 0,01029 | 0,03097 | 1,00 |
| C7-DC | acylcarnitines | 7,34 | 0,00134 | 0,01154 | 1,00 |
| C8 | acylcarnitines | 3,07 | 0,51456 | 0,5804 | 1,00 |
| C9 | acylcarnitines | 9,35 | 0,35227 | 0,42272 | 1,00 |
| Ala | aminoacids | 0,82 | 0,21978 | 0,28788 | 1,00 |
| Arg | aminoacids | 0,66 | 0,09341 | 0,14977 | 1,00 |
| Asn | aminoacids | 1,26 | 0,05594 | 0,10103 | 1,00 |
| Asp | aminoacids | 0,44 | 0,02596 | 0,05573 | 1,00 |
| Cit | aminoacids | 1,50 | 0,71279 | 0,77987 | 1,00 |
| Gln | aminoacids | 1,08 | 0,18057 | 0,24696 | 1,00 |
| Glu | aminoacids | 0,98 | 1 | 1 | 1,00 |
| Gly | aminoacids | 0,98 | 0,95669 | 0,97771 | 1,00 |
| His | aminoacids | 1,26 | 0,41559 | 0,48313 | 1,00 |
| Ile | aminoacids | 0,91 | 0,63536 | 0,69928 | 1,00 |
| Leu | aminoacids | 1,09 | 0,14282 | 0,20728 | 1,00 |
| Lys | aminoacids | 0,74 | 0,01598 | 0,04073 | 1,00 |
| Met | aminoacids | 0,75 | 0,26349 | 0,33114 | 1,00 |
| Orn | aminoacids | 0,74 | 0,1471 | 0,20728 | 1,00 |
| Phe | aminoacids | 0,99 | 0,87488 | 0,90909 | 1,00 |
| Pro | aminoacids | 0,64 | 0,00564 | 0,03097 | 1,00 |
| Ser | aminoacids | 0,73 | 0,09341 | 0,14977 | 1,00 |
| Thr | aminoacids | 0,77 | 0,05072 | 0,09435 | 1,00 |
| Trp | aminoacids | 0,99 | 0,87057 | 0,90909 | 1,00 |
| Tyr | aminoacids | 0,76 | 0,18057 | 0,24696 | 1,00 |
| Val | aminoacids | 1,09 | 0,7447 | 0,81002 | 1,00 |
| Ac-Orn | biogenic amines | 0,85 | 0,04196 | 0,08215 | 1,00 |
| ADMA | biogenic amines | 3,86 | 0,11788 | 0,17826 | 1,00 |
| alpha-AAA | biogenic amines | 0,94 | 0,00749 | 0,03097 | 1,00 |
| Carnosine | biogenic amines | 7,27 | 0,82814 | 0,88525 | 1,00 |
| Creatinine | biogenic amines | 0,87 | 0,56219 | 0,62242 | 1,00 |
| DOPA | biogenic amines |  |  |  | 0,00 |
| Dopamine | biogenic amines |  |  |  | 0,00 |
| Histamine | biogenic amines | 0,61 | 0,00399 | 0,02494 | 1,00 |
| Kynurenine | biogenic amines | 0,45 | 0,001 | 0,01154 | 1,00 |
| Met-SO | biogenic amines | 1,73 | 0,05594 | 0,10103 | 1,00 |
| Nitro-Tyr | biogenic amines | 0,00 | 1 | 1 | 1,00 |
| OH-Pro | biogenic amines | 1,00 | 1 | 1 | 1,00 |
| PEA | biogenic amines | 5,72 | 0,00025 | 0,00581 | 1,00 |
| Putrescine | biogenic amines | 1,12 | 0,1471 | 0,20728 | 1,00 |
| Sarcosine | biogenic amines | 2,36 | 0,00749 | 0,03097 | 1,00 |
| SDMA | biogenic amines | 1,27 | 0,79246 | 0,85201 | 1,00 |
| Serotonin | biogenic amines | 2,19 | 0,00402 | 0,02494 | 1,00 |
| Spermidine | biogenic amines | 1,64 | 0,11524 | 0,17826 | 1,00 |
| Spermine | biogenic amines | 1,50 | 1 | 1 | 1,00 |
| Taurine | biogenic amines | 1,42 | 0,11788 | 0,17826 | 1,00 |
| total DMA | biogenic amines | 1,17 | 0,05749 | 0,10283 | 1,00 |
| lysoPC a C14:0 | glycerophospholipids | 1,28 | 0,87488 | 0,90909 | 1,00 |
| lysoPC a C16:0 | glycerophospholipids | 0,87 | 0,11788 | 0,17826 | 1,00 |
| lysoPC a C16:1 | glycerophospholipids | 1,52 | 0,01099 | 0,03097 | 1,00 |
| lysoPC a C17:0 | glycerophospholipids | 0,95 | 0,87488 | 0,90909 | 1,00 |
| lysoPC a C18:0 | glycerophospholipids | 0,86 | 0,19273 | 0,25977 | 1,00 |
| lysoPC a C18:1 | glycerophospholipids | 1,33 | 0,003 | 0,02065 | 1,00 |
| lysoPC a C18:2 | glycerophospholipids | 1,04 | 0,32862 | 0,3969 | 1,00 |
| lysoPC a C20:3 | glycerophospholipids | 1,56 | 0,01099 | 0,03097 | 1,00 |
| lysoPC a C20:4 | glycerophospholipids | 1,53 | 0,07268 | 0,12393 | 1,00 |
| lysoPC a C24:0 | glycerophospholipids | 1,97 | 0,00749 | 0,03097 | 1,00 |
| lysoPC a C26:0 | glycerophospholipids | 3,67 | 0,00749 | 0,03097 | 1,00 |
| lysoPC a C26:1 | glycerophospholipids | 2,02 | 0,01069 | 0,03097 | 1,00 |
| lysoPC a C28:0 | glycerophospholipids | 2,29 | 0,00749 | 0,03097 | 1,00 |
| lysoPC a C28:1 | glycerophospholipids | 0,52 | 0,00136 | 0,01154 | 1,00 |
| PC aa C24:0 | glycerophospholipids | 18,71 | 0,01069 | 0,03097 | 1,00 |
| PC aa C26:0 | glycerophospholipids | 6,83 | 0,00749 | 0,03097 | 1,00 |
| PC aa C28:1 | glycerophospholipids | 1,76 | 0,00475 | 0,02758 | 1,00 |
| PC aa C30:0 | glycerophospholipids | 6,55 | 0,31319 | 0,38074 | 1,00 |
| PC aa C30:2 | glycerophospholipids | 0,10 | 0,00116 | 0,01154 | 1,00 |
| PC aa C32:0 | glycerophospholipids | 1,16 | 0,0297 | 0,06257 | 1,00 |
| PC aa C32:1 | glycerophospholipids | 1,37 | 0,03122 | 0,06381 | 1,00 |
| PC aa C32:2 | glycerophospholipids | 1,43 | 0,09341 | 0,14977 | 1,00 |
| PC aa C32:3 | glycerophospholipids | 2,06 | 0,02574 | 0,05573 | 1,00 |
| PC aa C34:1 | glycerophospholipids | 1,09 | 0,2116 | 0,28314 | 1,00 |
| PC aa C34:2 | glycerophospholipids | 0,87 | 0,07268 | 0,12393 | 1,00 |
| PC aa C34:3 | glycerophospholipids | 1,70 | 0,001 | 0,01154 | 1,00 |
| PC aa C34:4 | glycerophospholipids | 2,49 | 0,01099 | 0,03097 | 1,00 |
| PC aa C36:0 | glycerophospholipids | 0,87 | 0,05594 | 0,10103 | 1,00 |
| PC aa C36:1 | glycerophospholipids | 1,02 | 0,9135 | 0,9388 | 1,00 |
| PC aa C36:2 | glycerophospholipids | 0,86 | 0,00919 | 0,03097 | 1,00 |
| PC aa C36:3 | glycerophospholipids | 1,08 | 0,1471 | 0,20728 | 1,00 |
| PC aa C36:4 | glycerophospholipids | 1,04 | 0,3852 | 0,45346 | 1,00 |
| PC aa C36:5 | glycerophospholipids | 1,15 | 0,36763 | 0,43554 | 1,00 |
| PC aa C36:6 | glycerophospholipids | 3,35 | 0,01598 | 0,04073 | 1,00 |
| PC aa C38:0 | glycerophospholipids | 1,58 | 0,27737 | 0,34624 | 1,00 |
| PC aa C38:1 | glycerophospholipids | 0,59 | 0,00198 | 0,01472 | 1,00 |
| PC aa C38:3 | glycerophospholipids | 1,06 | 0,14282 | 0,20728 | 1,00 |
| PC aa C38:4 | glycerophospholipids | 1,20 | 0,21978 | 0,28788 | 1,00 |
| PC aa C38:5 | glycerophospholipids | 1,54 | 0,0005 | 0,01032 | 1,00 |
| PC aa C38:6 | glycerophospholipids | 1,12 | 0,04463 | 0,08471 | 1,00 |
| PC aa C40:1 | glycerophospholipids | 0,74 | 0,01069 | 0,03097 | 1,00 |
| PC aa C40:2 | glycerophospholipids | 1,21 | 0,02248 | 0,05074 | 1,00 |
| PC aa C40:3 | glycerophospholipids | 1,72 | 0,09248 | 0,14977 | 1,00 |
| PC aa C40:4 | glycerophospholipids | 0,87 | 0,23214 | 0,29984 | 1,00 |
| PC aa C40:5 | glycerophospholipids | 1,19 | 0,03122 | 0,06381 | 1,00 |
| PC aa C40:6 | glycerophospholipids | 1,00 | 0,41559 | 0,48313 | 1,00 |
| PC aa C42:0 | glycerophospholipids | 0,65 | 0,00025 | 0,00581 | 1,00 |
| PC aa C42:1 | glycerophospholipids | 0,71 | 0,00284 | 0,02029 | 1,00 |
| PC aa C42:2 | glycerophospholipids | 0,66 | 0,00774 | 0,03097 | 1,00 |
| PC aa C42:4 | glycerophospholipids | 1,60 | 0,15822 | 0,22127 | 1,00 |
| PC aa C42:5 | glycerophospholipids | 0,82 | 0,02248 | 0,05074 | 1,00 |
| PC aa C42:6 | glycerophospholipids | 1,51 | 0,02248 | 0,05074 | 1,00 |
| PC ae C30:0 | glycerophospholipids | 4,15 | 0,51487 | 0,5804 | 1,00 |
| PC ae C30:1 | glycerophospholipids | 12,83 | 0,00887 | 0,03097 | 1,00 |
| PC ae C30:2 | glycerophospholipids | 0,73 | 0,01063 | 0,03097 | 1,00 |
| PC ae C32:1 | glycerophospholipids | 1,23 | 0,26349 | 0,33114 | 1,00 |
| PC ae C32:2 | glycerophospholipids | 2,38 | 0,79246 | 0,85201 | 1,00 |
| PC ae C34:0 | glycerophospholipids | 1,40 | 0,26349 | 0,33114 | 1,00 |
| PC ae C34:1 | glycerophospholipids | 1,24 | 0,001 | 0,01154 | 1,00 |
| PC ae C34:2 | glycerophospholipids | 0,99 | 0,31319 | 0,38074 | 1,00 |
| PC ae C34:3 | glycerophospholipids | 1,69 | 0,04416 | 0,08471 | 1,00 |
| PC ae C36:0 | glycerophospholipids | 2,19 | 0,1471 | 0,20728 | 1,00 |
| PC ae C36:1 | glycerophospholipids | 1,06 | 0,42782 | 0,48819 | 1,00 |
| PC ae C36:2 | glycerophospholipids | 0,99 | 0,91356 | 0,9388 | 1,00 |
| PC ae C36:3 | glycerophospholipids | 1,55 | 0,1471 | 0,20728 | 1,00 |
| PC ae C36:4 | glycerophospholipids | 1,31 | 0,0146 | 0,03934 | 1,00 |
| PC ae C36:5 | glycerophospholipids | 1,25 | 0,02248 | 0,05074 | 1,00 |
| PC ae C38:0 | glycerophospholipids | 1,52 | 0,00175 | 0,01414 | 1,00 |
| PC ae C38:1 | glycerophospholipids | 1,08 | 0,09341 | 0,14977 | 1,00 |
| PC ae C38:2 | glycerophospholipids | 0,97 | 0,87488 | 0,90909 | 1,00 |
| PC ae C38:3 | glycerophospholipids | 1,06 | 0,1286 | 0,19291 | 1,00 |
| PC ae C38:4 | glycerophospholipids | 1,32 | 0,00475 | 0,02758 | 1,00 |
| PC ae C38:5 | glycerophospholipids | 1,18 | 0,01598 | 0,04073 | 1,00 |
| PC ae C38:6 | glycerophospholipids | 1,35 | 0,02585 | 0,05573 | 1,00 |
| PC ae C40:1 | glycerophospholipids | 1,05 | 0,18057 | 0,24696 | 1,00 |
| PC ae C40:2 | glycerophospholipids | 0,83 | 0,23214 | 0,29984 | 1,00 |
| PC ae C40:3 | glycerophospholipids | 0,81 | 0,21978 | 0,28788 | 1,00 |
| PC ae C40:4 | glycerophospholipids | 1,11 | 0,03416 | 0,06832 | 1,00 |
| PC ae C40:5 | glycerophospholipids | 1,21 | 0,07287 | 0,12393 | 1,00 |
| PC ae C40:6 | glycerophospholipids | 1,00 | 0,35584 | 0,42428 | 1,00 |
| PC ae C42:0 | glycerophospholipids | 1,85 | 0,0146 | 0,03934 | 1,00 |
| PC ae C42:1 | glycerophospholipids | 1,91 | 0,01686 | 0,04238 | 1,00 |
| PC ae C42:2 | glycerophospholipids | 0,80 | 0,31319 | 0,38074 | 1,00 |
| PC ae C42:3 | glycerophospholipids | 1,24 | 0,42782 | 0,48819 | 1,00 |
| PC ae C42:4 | glycerophospholipids | 0,70 | 0,001 | 0,01154 | 1,00 |
| PC ae C42:5 | glycerophospholipids | 0,93 | 0,02994 | 0,06257 | 1,00 |
| PC ae C44:3 | glycerophospholipids | 1,80 | 0,01099 | 0,03097 | 1,00 |
| PC ae C44:4 | glycerophospholipids | 0,43 | 0,00025 | 0,00581 | 1,00 |
| PC ae C44:5 | glycerophospholipids | 1,65 | 0,42782 | 0,48819 | 1,00 |
| PC ae C44:6 | glycerophospholipids | 0,93 | 0,30246 | 0,37505 | 1,00 |
| SM (OH) C14:1 | sphingolipids | 1,57 | 0,00749 | 0,03097 | 1,00 |
| SM (OH) C16:1 | sphingolipids | 2,78 | 0,00025 | 0,00581 | 1,00 |
| SM (OH) C22:1 | sphingolipids | 2,45 | 0,04463 | 0,08471 | 1,00 |
| SM (OH) C22:2 | sphingolipids | 1,15 | 0,04196 | 0,08215 | 1,00 |
| SM (OH) C24:1 | sphingolipids | 8,13 | 0,02248 | 0,05074 | 1,00 |
| SM C16:0 | sphingolipids | 1,76 | 0,00025 | 0,00581 | 1,00 |
| SM C16:1 | sphingolipids | 1,14 | 0,11788 | 0,17826 | 1,00 |
| SM C18:0 | sphingolipids | 1,44 | 0,00198 | 0,01472 | 1,00 |
| SM C18:1 | sphingolipids | 1,28 | 0,01075 | 0,03097 | 1,00 |
| SM C20:2 | sphingolipids | 1,38 | 0,01099 | 0,03097 | 1,00 |
| SM C22:3 | sphingolipids | 0,82 | 0,56219 | 0,62242 | 1,00 |
| SM C24:0 | sphingolipids | 1,32 | 0,56219 | 0,62242 | 1,00 |
| SM C24:1 | sphingolipids | 1,69 | 0,07329 | 0,12393 | 1,00 |
| SM C26:0 | sphingolipids | 1,45 | 0,02248 | 0,05074 | 1,00 |
| SM C26:1 | sphingolipids | 1,73 | 0,07268 | 0,12393 | 1,00 |
| H1 | sugars | 9,65 | 0,11788 | 0,17826 | 1,00 |
